# Assessing the methodological quality of systematic reviews

**DOI:** 10.1038/npjpcrm.2015.19

**Published:** 2015-03-19

**Authors:** Emma J Welsh, Rebecca A Normansell, Christopher J Cates

**Affiliations:** 1 Population Health Research Institute, St George's University of London, London, UK

Dear Sirs,

We read with interest the paper by Ho *et al*,^1^ which used the AMSTAR tool to assess the methodological quality of systematic reviews (SRs) on chronic obstructive pulmonary disease (COPD). As staff at the Cochrane Airways Group with the responsibility of producing high-quality SRs for airway conditions, including COPD, we are always happy to hear how we could improve.

However, there are some methodological issues within the study. The abstract states that the methodological quality of the reviews was disappointing and emphasises the more negative findings, neglecting the positive results (e.g.,* a priori* design in 67% SRs, comprehensive literature search in 97% and scientific quality assessed and documented in 85%). The authors did not complete the AMSTAR ratings in duplicate; yet, duplicate data extraction is a mark of a good SR. Our experience with this tool is that the discussion between two or more people helps reach a fair judgement.^2^ It would have been helpful to see the AMSTAR ratings per review so that the work could be replicated and evaluated.

We noted the lack of discussion about the choice to limit the study to SRs that include a meta-analysis. Choosing not to perform a meta-analysis when there is a lot of heterogeneity between studies is a valid decision.

The authors highlighted that non-English databases were searched infrequently. This is not an AMSTAR criterion; have the authors suggested that this be incorporated in any update of the tool? Cochrane does not require that non-English language databases be searched and this is usually done only when we expect that this will yield additional relevant trials. We agree that multilingual SR teams are advantageous and we would be grateful if people who wish to translate the trial reports for inclusion in Cochrane reviews contact us.

We take the authors’ point about being clearer about reviewers’ support, and making a statement about publication bias in the results section as well as the methods section when there are too few studies to permit a funnel plot.

As highlighted in the paper, the quality of SRs has improved significantly in recent years through the development of methods and improved implementation.^3,4^ It would have been helpful to highlight this important point in the conclusions and abstract.

## Notes

We are employees of the Cochrane Airways Group with editorial responsibility for the reviews that are published. EW and CC have authored Cochrane reviews included in this analysis. The Cochrane Airways Group is a part of the international Cochrane Collaboration, an independent not-for-profit consortium dedicated to providing up-to-date accurate information about the effects of health care. The National Institute for Health Research (NIHR) is the largest single funder of the Cochrane Airways Group.
